# Remote Blood Glucose Monitoring in *mHealth* Scenarios: A Review

**DOI:** 10.3390/s16121983

**Published:** 2016-11-24

**Authors:** Giordano Lanzola, Eleonora Losiouk, Simone Del Favero, Andrea Facchinetti, Alfonso Galderisi, Silvana Quaglini, Lalo Magni, Claudio Cobelli

**Affiliations:** 1 Department of Electrical, Computer and Biomedical Engineering, University of Pavia, 27100 Pavia, Italy; eleonora.losiouk01@ateneopv.it (E.L.); silvana.quaglini@gmail.com (S.Q.); 2 Department of Information Engineering, University of Padova, 35131 Padova, Italy; sdelfave@dei.unipd.it (S.D.F.); andrea.facchinetti@dei.unipd.it (A.F.); claudio.cobelli@unipd.it (C.C.); 3 Neonatal Intensive Care Unit, Department of Woman and Child’s Health, University of Padova, 35131 Padova, Italy; alfonsogalderisi@gmail.com; 4 Department of Civil Engineering and Architecture, University of Pavia, 27100 Pavia, Italy; lalo.magni@unipv.it

**Keywords:** blood glucose sensors, remote monitoring, *mHealth*, Internet of Things (IoT) in health care

## Abstract

Glucose concentration in the blood stream is a critical vital parameter and an effective monitoring of this quantity is crucial for diabetes treatment and intensive care management. Effective bio-sensing technology and advanced signal processing are therefore of unquestioned importance for blood glucose monitoring. Nevertheless, collecting measurements only represents part of the process as another critical task involves delivering the collected measures to the treating specialists and caregivers. These include the clinical staff, the patient’s significant other, his/her family members, and many other actors helping with the patient treatment that may be located far away from him/her. In all of these cases, a remote monitoring system, in charge of delivering the relevant information to the right player, becomes an important part of the sensing architecture. In this paper, we review how the remote monitoring architectures have evolved over time, paralleling the progress in the Information and Communication Technologies, and describe our experiences with the design of telemedicine systems for blood glucose monitoring in three medical applications. The paper ends summarizing the lessons learned through the experiences of the authors and discussing the challenges arising from a large-scale integration of sensors and actuators.

## 1. Introduction

Tight monitoring of blood glucose levels is of capital importance for keeping this key physiological parameter under control, and is needed in a number of medical applications encompassing the treatment of diabetes and the management of intensive care therapy, which are briefly described in the following.

Diabetes is a family of chronic diseases affecting an ever increasing number of patients. According to the World Health Organization, the worldwide prevalence of diabetes was 422 million adults in 2014, increasing from 108 million in 1980 [1]. It is classified into three major disease types [2] as follows: Type 1 Diabetes (T1D) [3] is caused by the destruction of the beta-cells in the pancreatic islets of Langerhans where insulin is produced and secreted; Type 2 Diabetes (T2D) is caused either by a decreased insulin secretion by the pancreas or by some kind of “resistance” to insulin developed by the body [4]; and finally, Gestational Diabetes (GD) may arise during pregnancy in 2%–6% of cases and increase the risk of complications for both mother and child before and after childbirth [5]. All diabetes types cause an abnormal increase in Blood Glucose Level (BGL). In particular, prolonged hyper-glycemia leads to metabolic unbalance and is associated with long-term macro-vascular complications such as stroke or coronaropathy and micro-vascular ones such as nephropathy, neuropathy or retinopathy. Despite the lack of a definitive healing treatment for diabetes, new drugs and devices are helping patients in the challenging task of maintaining an adequate glucose control, easing the disease management, and improving their quality of life. Moreover, a tighter glucose monitoring results in a more effective therapeutic strategy that eventually yields a more significant reduction in the complications associated with diabetes [6]. Therefore, in the past few decades, the development of glucose sensors saw an impressive boost, whose most prominent achievement is given by the commercial availability of subcutaneous sensors for Continuous Glucose Monitoring (CGM) that are becoming a realistic option for home-monitoring and might soon replace the traditional point-of-care BGL measurement [7,8]. For instance, recently, the U.S. Food and Drug Administration panel expressed a positive opinion about the use of the *Dexcom G5* CGM sensor as a substitute for glucometers based on fingerstick tests [9].

Avoiding hyper- and hypo-glycemia is very important also in critically ill patients, such as those admitted in intensive care units. In these cases, the need arises to adopt a suitable algorithm for the regulation of glucose infusion based on CGM to improve glycemia control and prevent large BGL oscillations as well as hypo- or hyper-glycemia episodes. For example, failure to achieve a proper glucose control in preterm newborns is associated with an impaired neurological development only observed during the second year of life [10], while in the short-term, there is a worsening of clinical outcomes in terms of mortality, infections and brain hemorrhage [11,12]. Thus, CGM represents an improvement in the standard therapy in which the actual detection of hypo- and hyper-glycemia episodes is difficult, as they may happen between readings.

Effective sensing technology and advanced signal processing techniques are certainly of capital importance to attain an effective blood glucose monitoring. Nevertheless, collecting measurements only represents part of the process, since other essential tasks are delivering these measures and/or any related alerts to the several actors involved in the blood-glucose management process. Those actors include the clinical staff responsible for planning and administering the therapy, the parents of pediatric patients or the significant others of adult ones. Despite being located far from the monitored patient, these people need to be kept up to date with his/her conditions in order to undertake the most appropriate actions. A remote monitoring system, by taking care of the delivery of any relevant information to the right players, becomes an important part of the sensing architecture.

In this paper, we emphasize the advantages of remote monitoring, illustrating the technological components required to implement it. We start recalling the evolution that occurred within the Information and Communication Technology (ICT) architectures for acquiring data from patients and sending them remotely for perusal or analysis. Although we provide examples mainly concerning glucose monitoring or diabetes management, the applicability is rather general as they may be effectively exploited in different clinical contexts as well. We also illustrate some projects carried on by the authors where glucose sensors and insulin pumps have been integrated into a remote monitoring architecture operating in real time. Differences will be highlighted among systems addressing various patient populations, such as adults, children and newborns. The paper ends with a discussion on the lessons learned by the authors and the current trends for deploying remote monitoring solutions on a large scale.

## 2. The Evolution of the Architectures for Remote Monitoring

Even though remote monitoring has not entered the routine clinical practice yet, during the past few decades, several projects have been developed in different clinical contexts exploiting it for research purposes. During this time period, the dramatic progress occurred in ICT has deeply affected the way in which remote monitoring is accomplished. In the following section, we review the evolution of the main architectures that have been used over the years to collect data from patients, with particular emphasis on glucose and diabetes monitoring context.

### 2.1. The Early Remote Monitoring Architecture Adopted for Diabetes Patients (1990–2000)

**Figure 1:**
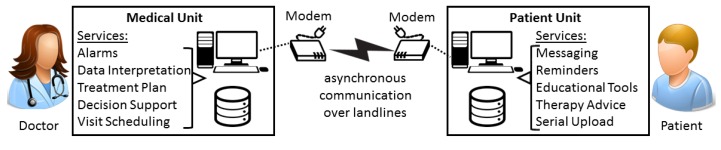
The early architecture adopted for the remote monitoring of Type 1 Diabetes Patients. The architecture encompassed two separate units, each one hosting different services, asynchronously communicating using modems operating over landlines.

The first prototypical systems attempting a systematic collection of data from diabetes patients started to appear in the late 1990s [13]. The limitations of the technology and the brittleness of the network connections available at that time accounted for an architecture encompassing two separate elements, a Patient Unit (PU) and a Medical Unit (MU) that provided different services to those two user classes [14]. The PU was located at the patient’s home and was implemented as an application usually running on a Personal Computer (PC). Its main purpose was data acquisition, which was mostly accomplished manually by the patient himself, even though sometimes it also occurred automatically by interfacing the PU with sensing devices [15]. The MU acted as a concentrator for patient data and was located instead at the clinic in order to be always accessible by the treating staff for consultation. The PU and MU exchanged data asynchronously through network connections established by modems over regular telephone lines, as shown in Figure 1. Sometimes the PU, or part of it, was also implemented on a Personal Digital Assistant (PDA). These were small portable devices with limited computational and no networking capabilities that had to be placed on a docking station wired to a PC for accomplishing any network operation. As such, they privileged mobility and availability with respect to connectivity. Nevertheless, since the whole connectivity model was inherently asynchronous and real-time monitoring was not affordable at that time, at least in the beginning, the tradeoff seemed to be reasonable.

The strict separation between the PU and MU was dictated by the discontinued nature of the connection and the need to render each component independent from the other one. This separation also allowed additional services to be easily integrated into each component since both stored patient data locally. As a matter of fact, it soon became clear that, for remote monitoring systems to be effective, besides just offering plain data collection, they had to show a proactive behavior processing those data at the knowledge level [16,17,18]. Thus, on the patient side, additional services were introduced addressing self-management such as reminders, calculators for selecting each time the proper amount of insulin to be administered, comprehensive charts providing a better overview of the disease or even educational tools [19]. On the doctor’s side, services for promptly recognizing and managing abnormal patient conditions were devised instead.

### 2.2. The Switch to a Service Oriented Architecture (2000–2010)

**Figure 2:**
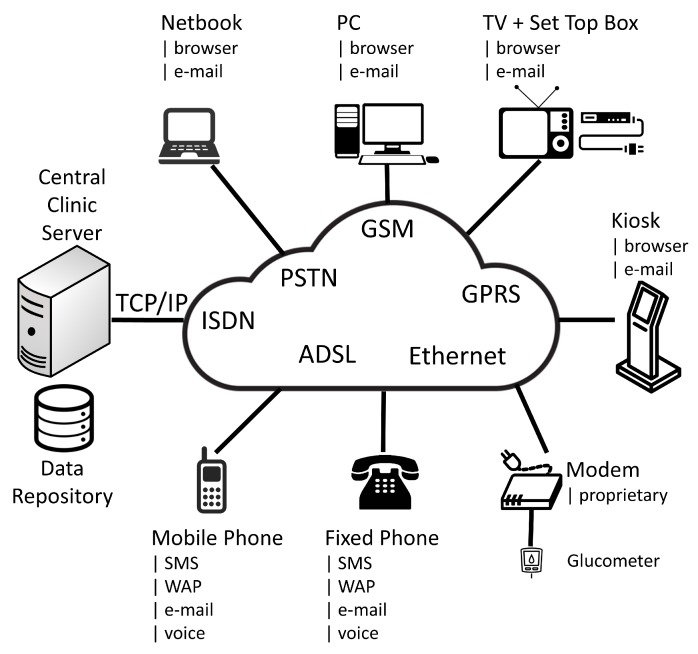
The web-centric architecture emerged during the first decade of the new century. A large number of devices accessed a central server using multimodal techniques.

The strong distinction between PU and MU faded during the first decade of the new century, as telemedicine services began to be deployed over stable network connections, eventually accessed through mobile devices such as smartphones [20] that were just appearing on the market. The previous partitioning into two opposite halves, each one targeting a specific set of functionalities did not provide the required flexibility anymore. Instead, the switch to a basic client-server paradigm, such as the one used by plain web applications, granted ubiquitous access to a set of centralized services, resulting in a Service Oriented Architecture (SOA) [21]. The SOA also allowed the rapid deployment of new services, avoiding the need to undergo complex installations or configurations at every patient’s location. Moreover, leveraging the interoperability among the components, it also favored a multi-modal access exploiting different technologies that were emerging at that time, such as Short Message Service (SMS), Wireless Application Protocol (WAP), automatic speech recognition or TV set-top boxes, as shown in Figure 2.

The switch to the SOA paralleled the PDAs gradual demise. In fact, since PDAs were mainly connectionless devices, they could not stand the widespread availability of network connections. However, despite smartphones were hailed as the successors of the PDAs, during the first decade of the current century, they had very poor computational power and limited display capabilities that made an immediate replacement of PDAs almost impossible. Thus, it was not unusual to observe some blending of SOA with older architectures depending on the devices used [22].

The early versions of the *Diabeo* system [23], for example, still used a PDA to implement a logbook where the patient kept track of all his BGL readings. The PDA also offered a decision support system automatically proposing prandial and basal insulin dosages. However, every other functionality was made available through a web application that could be accessed anywhere using PCs or other appliances able to run a web browser. The *M^2^DM* project [24] represented instead a step forward since it was based on a pure SOA architecture, and the interfacing of glucometers was accomplished using the *Roche Acculink* system. Despite the latter was a closed system using a proprietary modem technology, it simplified the remote transfer of patient data fully preserving the SOA paradigm in that project. Incidentally, we may note that glucose sensors lag far behind current ICT standards usually found in other appliances, and, in most cases, they are not compliant with those standards at all. As a matter of fact, across the first decade of the century, glucometers even lacked basic Universal Serial Bus (USB) or wireless capabilities and their interfacing occurred just through serial connections or dedicated infrared transceivers hooked up to the PC serial port. Eventually, by the end of the first decade, some projects began to use smartphones for encapsulating specific services on the patient’s side, such as alerts and reminders [25], therefore complementing the SOA as it happened in the *LifePhone* project [26].

### 2.3. The Evolution to a Body Area Network Architecture (2010–Present)

**Figure 3:**
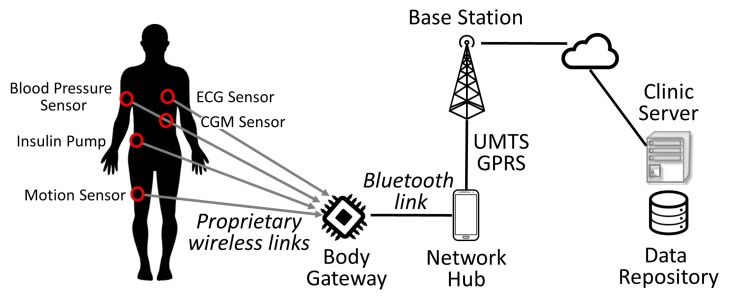
The architecture adopted for a Body Area Network sees multiple wearable devices coordinated by a *Body Gateway*. The *Body Gateway* interacts using *Bluetooth* with the *Network Hub* that enacts remote monitoring.

Rather interestingly, the improvements in ICT caused once more a paradigm shift concerning data collection and upload. The need to acquire signals using multiple sensors attached to the body, and possibly operate on it through different actuator devices led to a new architectural schema known as Body Area Network (BAN) [27]. BANs are wireless networks having a very limited range and interconnecting several small devices usually worn by the patient that are located within a distance of just a couple of meters. In this case, directly connecting each single device to the cloud is impossible for many reasons. First, these devices have very limited size and sometimes are even implanted under the patient skin, posing severe limitations both on their functional capabilities and power source availability. Moreover, similar to mobile phones, the radio emission required to connect to a baseband station using Third Generation Mobile Communication System (3G)/Universal Mobile Telecommunications System (UMTS) is on the order of 500 to 3000 mW and is definitely unacceptable for devices operating so close to the human body [28]. Finally, depending on the purpose of the BAN, an actuator may be involved, such as an insulin pump or a cardioverter device, and the logic for controlling them must be part of the very same BAN to avoid delays or unresponsive events in case of poor connectivity. Thus, a dedicated component coordinating all the devices is required such as the *Body Gateway* shown in Figure 3. This component interacts using *Bluetooth* with the *Network Hub*, usually a smartphone, which is the key for enabling network connectivity and enacting the remote monitoring that is the subject of this paper. A BAN architecture may be instantiated with minimal work to support several scenarios exploiting different devices in various medical contexts [29,30].

Incidentally, we observe that the computational power of modern *Android*^TM^ smartphones is suitable for running even complex algorithms. Moreover, smartphones may also be effectively used as functional input devices thanks to their valuable displays besides still acting as *Network Hubs* for the telemedicine services. Thus, they are being increasingly used as prototyping devices with enhanced graphic capabilities that are available at a cost much lower than the one required for designing a custom new board from scratch. When a smartphone is used according to this paradigm, besides acting as a *Network Hub*, it also plays the role of a *Body Gateway*, with both components coalescing into a single device. However, when this solution is implemented, all the body connected devices must be capable of communicating using *Bluetooth* technology, since this is the only short range wireless technology supported by smartphones. An interesting example is given by the *Diabetes Assistant (DiAs)* [31,32], which is basically a commercial *Android*^TM^ smartphone used for implementing an Artificial Pancreas. The *DiAs* will be better described in Section 4.1 and Section 4.2, since it has been used by the authors in two studies involving remote monitoring on diabetes patients.

## 3. Sending Data from Diabetes Devices to the Cloud

The architectures for remote monitoring have evolved over the years, as described in Section 2. This evolution closely paralleled the improvements in the control logic of the devices generating the data to be monitored, as their manufacturers increasingly added computational and connectivity capabilities over time, leveraging the continuous achievements in ICT and microelectronics. The very first glucometers were analog devices able to acquire just one measurement at a time [33]. The switch to digital technology allowed for retaining several measurements in memory, while the parallel massive diffusion of the PC favored their interfacing with the devices and paved the way for remote monitoring applications. At first, manufacturing companies only allowed downloading data from their devices using serial ports and offered logbook applications to better analyze data on the PC display using charts and diagrams. These applications were used either by specialists that downloaded data from their patients’ glucometers at regular visits, or by technology-savvy patients that annotated BGL readings with additional information for an improved control. *Roche Diabetes Care GmbH* (Mannheim, Germany) was the first company to sell a complete remote monitoring system with their *Acculink*. This was essentially a proprietary modem that allowed patients to regularly send data from their *Accu-Chek* glucometers to their doctor’s office even without owning a PC. *Roche* also provided the application to be installed on a server at the clinic for receiving and storing the patient’s data, therefore ensuring a better follow-up by doctors among scheduled visits [34].

Currently, almost any manufacturer supports downloading data from their glucometers. However, this still happens through different proprietary protocols that prevent the actual integration of data into a single application. Moreover, manufacturing companies are not interested in running remote monitoring services by themselves. Those problems were recognized by *DiaSend* (Askim, Sweden), which started its business in 2006 offering integration capabilities for all the major glucometers on the market in addition to many insulin pumps. The patient may use either his PC or his smartphone to download data from the devices and upload them onto a remote server managed by *DiaSend* so that they become easily accessible to the clinical staff. The company claims that almost 2000 clinics worldwide have already made arrangements with them and nearly 350,000 patients are regularly using it.

Smartphones have been also increasingly exploited by manufacturers to simplify the acquisition of BGL measurements and import their values into the digital ecosystem. An interesting example is provided by *iBGSTAR^®^* produced by *Sanofi* (Paris, France), which is essentially a glucometer implemented as a hardware plugin for the smartphone. It is a very small piece of hardware that fits the *iPhone* or *iPod* docking connector and allows for measuring BGL using test strips. The hardware has a very small display that comes in handy just to preserve its usability as a standalone tool. However, the *iBGSTAR^®^* is best used in combination with the *Diabetes Manager* app, also from *Sanofi*. This app allows for importing BGL readings into the smartphone where they may be complemented with additional information manually provided by the patient concerning meals, insulin delivery, physical activity, drugs taken or notes. The application gives then a complete overview of the patient’s state since it allows searching for specific events and produces charts and statistic diagrams.

A different remote monitoring paradigm is being adopted by *Dexcom Inc.* (San Diego, CA, USA) with their *G5 Mobile CGM System*. This system encompasses two different smartphone applications, the *Mobile/Share App* and the *Follow App*. The former is installed on the patient smartphone and displays real-time glucose data and trends directly. Using this app, the patient may designate up to five people as followers, thereby including caregivers, clinical staff, family members or significant others. Once those people have installed the *Follow App* on their smartphones, they will all receive in real time the CGM readings and will be notified about critical situations occurring to the patient in order to possibly undertake actions.

The need for a monitoring platform that overcomes the inherent limitations of proprietary protocols is probably best witnessed by the *Nightscout Project* [35], which was initially born as *CGM in the Cloud* and eventually turned into *The Nightscout Foundation* [36] in 2014. This is an open source project started by some parents of children affected by T1D that are implementing a platform for real-time access to the patients’ CGM data via websites, smartphones or smartwatches for their own serenity. Currently, *Nightscout* is able to interface and acquire data from sensors manufactured by *Dexcom* (*G4, G4 with Share, G5*), *Medtronic* (*640g, 530g, Veo*) and *Abbott* (*FreeStyle Libre*).

## 4. Our Experiences with Telemedicine Systems

During the past several years, the authors have been involved in the development of several telemedicine systems. These systems have been used in important clinical trials for remotely overseeing blood-glucose control. In this section, we summarize our experiences focusing on three projects, each one having different features and, in particular, different recipients of the information generated by the blood-glucose measurement devices.

### 4.1. Artificial Pancreas in Adults

Since 2007, our group has been active in the design and testing of a closed-loop strategy for the treatment of T1D in adult patients, the so-called Artificial Pancreas (AP). The AP [37] is a minimally-invasive device for the automatic glycemic control in T1D, modulating insulin infusion based on the measurements collected by a real-time CGM sensor. An AP encompasses at least three components tightly interconnected: a CGM sensor for continuously acquiring BGL readings; an insulin pump for delivering insulin boluses to the patient and a control algorithm driving the pump based on the readings acquired in real time from the CGM sensor. Additional components are usually represented by an interaction display, which is used by the patient to enter information used by the algorithm, such as meal intakes, manual BGL readings for calibrations or for commanding the extra boluses needed to compensate for the meals.

Since 2010, we have been part of the *AP@home* project, an initiative funded by the European Union within the 7th Framework Programme aimed at designing and implementing the AP [38]. During the five-year project lifespan, several clinical trials were planned, enrolling patients over longer times and scheduling their experiments in environments getting each time closer to real-life conditions. The first experiments were conducted in 2011 on hospitalized patients under strict protocol prescriptions with the aim of enforcing the highest level of safety on them. In this highly controlled set-up, the duration of each experiment was limited to just one day including the overnight stay [39]. The study team (at least one clinician and one bioengineer) was constantly attending the experiment, limiting the need for a telemedicine system. Nevertheless, a first prototype version of the remote monitoring system was designed [40], and eventually deployed and tested [41]. This first study proved that the AP was safe in a controlled environment, and thus we moved our research towards testing in less controlled environments, more closely resembling real-life [42,43]. These studies, lasting two days, were held in a hotel where both the study team and the patients were lodged. Home use of the device was simulated with the study team minimizing the interaction with the patient and intervening only to solve technical or medical issues. The availability of a telemedicine system, making available to the study team the blood-glucose measures and any other medical or technical information [44], was fundamental for the design of the study. This system guaranteed the highest level of safety during the simulation of independent AP use and helped in obtaining the study approval by the Ethics Committees. In fact, during the first hospital trial [42], the brittleness of the connection between the AP and the sensor caused frequent disconnections that occurred particularly overnight when the patient was asleep [45]. However, the remote monitoring system signaled those problems to the staff that could promptly intervene fixing them and avoiding any risk for the patients. Eventually, in 2014–2015, a large trial was scheduled testing the use of the AP at home for two months in 30 adult patients [46]. Out of these patients, 20 subjects extended the AP use for one extra month [47]. Also in this case, the telemedicine system and the possibility to oversee the experiments were crucial to providing the required remote assistance during the trial.

Throughout all of these trials, the main users of the telemedicine system were the members of the clinical team and the bioengineers overseeing the study. The effectiveness of the remote monitoring system was assessed after each trial, resulting in a close collaboration among clinicians and bioengineers, with the goal of improving it. Over time, summary panels were added to allow a prompt recognition of the situation by the staff; visual and audible alarms were implemented to immediately draw user attention whenever required; and the detailed patient chart was improved tracking additional events such as exercises, stress tests, notes, etc. Eventually, an additional component was developed sending summary messages or alarms to the staff phones, which were used as pagers. This was particularly useful during longer trials scheduled near the end of the project, when the supervision by the staff around the clock was no longer affordable.

Although, for the *AP@home* project, the main users of the telemedicine system remained the members of the study team, we took the opportunity to investigate the patient perception of the usefulness of that tool. For this purpose, we administered ad hoc questionnaires about the assistance time required in case of complications, the quality of patient–physician communication, the reduction of control visits in terms of number and costs, the perceived serenity and safety, and the amount of money that patients would be willing to pay for the system. In nearly 80% of the answers, patients highlighted positive feelings towards the service. However, after comparing questionnaires administered during the hotel trials with those occurring at home, it emerged that, in the latter case, patients were more uncertain about the usefulness of the system. The increment in the percentage of answers expressing uncertainty, increasing from 3% at the hotel to 17% at home, could be due to the need of being more tightly monitored perceived by the patients when they are at home.

**Figure 4:**
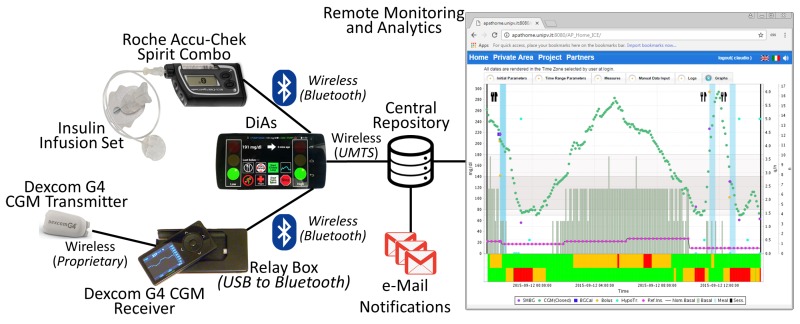
The architecture illustrating the components used in the final trial of *AP@home*, with the *Diabetes Assistant* acting both as a *Body Gateway* and as a *Network Hub*.

The hardware used for remote monitoring was upgraded several times during the *AP@home* project development, paralleling the improvements on the AP, to reach the level of functionality and reliability required for the main trial at home. A detailed description of that evolution and the performance of each hardware configuration adopted is provided in [45]. The final configuration used in *AP@home* is reported in Figure 4 and sees the *DiAs* [31] as the core component playing both the roles of *Body Gateway* and *Network Hub*.

The *DiAs* is a smartphone-based AP platform capable of running the control algorithm. To ensure the operation of the smartphone as a medical device, the Linux kernel of its operating system (i.e., *Android*^TM^) was modified to remove any application not related to the study; prevent the installation of external applications from the store; forbid its use for issuing voice calls or surfing the web; and include self-checks concerning system integrity. The smartphone adopted for the study was a *Nexus 5* by *LG Electronics* (Seoul, Korea) offering state-of-the-art *Bluetooth* wireless connectivity that was used for directly interacting with the peripherals: the CGM sensor and the insulin pump. Thus, according to this configuration, the smartphone acted as *Body Gateway*.

While the availability of state-of-the-art technology is usually desirable, it is often a bottleneck when dealing with medical devices. In this study, the insulin pump of choice was the *Roche Accu-Chek Spirit Combo* by *Roche Diagnostics GmbH* (Mannheim, Germany), that natively offered the possibility of being wirelessly controlled using *Bluetooth*. However, the CGM of choice was the *Dexcom G4 Platinum* by *Dexcom, Inc.* (San Diego, CA, USA), which only provided a USB port and lacked *Bluetooth* connectivity. To overcome this problem, a USB to *Bluetooth Low Energy* converter (i.e., the *Relay Box* in Figure 4) was developed and employed in combination with the *Dexcom G4 Platinum*. The *DiAs* then acted as a *Network Hub* sending all the information acquired by the peripherals or generated internally by the AP controller to the remote monitoring clinic server using a *3G/UMTS* connection in real time. Once on the clinic server, these data became eventually accessible through the web according to a standard SOA architecture for remote monitoring or analytic purposes [41].

### 4.2. Artificial Pancreas in Adolescents

Since 2015, our AP system has been tested mostly in adult patients and only one experiment was performed on other populations: namely a one-day study on hospitalized adolescents [48]. Stimulated by the highly positive results achieved during the *AP@home* project, we decided to investigate the use of the AP also in pediatric subjects, starting from prepubertal children at school age. Unlike adolescents that were the subject of more than 19 studies [49], this fragile population has received relatively little attention in the AP literature due to its very challenging nature. Thus, in 2015, we conducted a clinical trial testing our AP system on 30 children, 5–9 years old, and compared its performance with the traditional micro-infusion pump therapy manually operated by the patient’s parents. The study was conceived as a randomized crossover trial, that took place during a summer camp in a resort village in Bardonecchia, a small city located in northern Italy. Patients were enrolled in five different major centers in Italy. As soon as the patients were admitted at the camp, they were randomly assigned to one of the two arms: one arm used the AP in the first part of the study and the traditional manually operated pump in the second, while the other arm did the opposite. Each part lasted for three days (72 h) and the two were separated by a one-day wash-out period [50].

**Figure 5:**
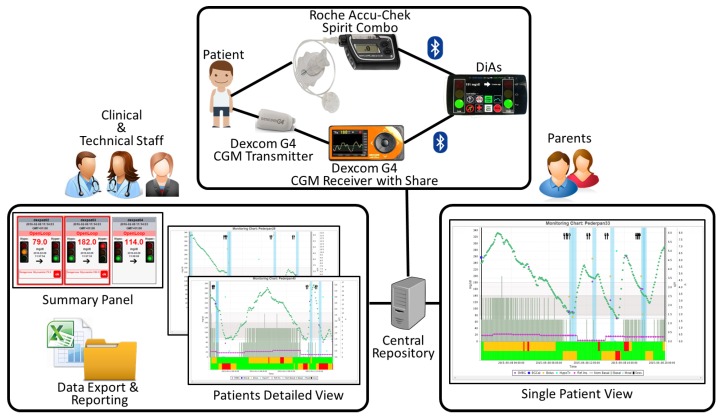
The remote monitoring architecture used for the adolescents camp held in Bardonecchia. The system was used by the clinical and technical staff for safety and research purposes and was proposed to the parents as a means of managing the disease of their children.

The architecture adopted for the adolescent camp was almost the same as that already adopted for *AP@home*, with the difference that this time the *Dexcom G4 Platinum with Share* was used as a CGM sensor. This version of the *Dexcom G4* natively offered *Bluetooth Low Energy* connectivity, thus eliminating the need for the additional *USB to Bluetooth* converter and enhancing the robustness of the system. The architecture is shown in Figure 5. In this project, the telemedicine system served a double role: on one hand, it was used by the study team to supervise the study, especially overnight, exactly as it happened during *AP@home*. On the other hand, it was also used by the parents, especially those who were not attending the camp, to remotely monitor their children. Indeed, it is known from the literature that the unawareness of the BGL of their children is the cause of great concern in parents [51], especially for children in the age range of those enrolled by our study. This is eloquently testified by the success of the Nightscout project, previously mentioned in Section 3.

To this aim, the remote monitoring system had been updated in order to allow different access levels depending on the role of its users. The members of the staff had complete and unrestricted access to all the subjects enrolled in the trial. Thus, they could access all the summary panels containing the alarms as well as the detailed views of any patient, including the possibility of manually updating the information through the web and downloading data. The parents of each patient were allowed instead to access a read-only view only reporting the real-time situation of their child, and were denied the possibility of modifying any data. To assess the parents’ perception of the remote monitoring system, we administered some questionnaires before and after the trial. Even though parents could use the system just for the very limited period of the study, the survey showed that they would be willing not only to use it regularly but also to pay a fee for that.

### 4.3. Neonatal Intensive Care Unit

The last project developed by the authors early in 2016 and still under way is *Neokid*. *Neokid* originated from the need to tightly control blood glucose in preterm newborns admitted at the Neonatal Intensive Care Unit (NICU) of the University Hospital in Padova. Since preterm newborns are fragile patients, heel pricks for taking blood samples may not be as frequent as required, and the probability that hypo- or hyper-glycemia episodes will go undetected is much higher than in adults. To overcome the problem, the NICU started investigating the effectiveness of using CGM sensors to support the best practices for calculating glucose infusion rates based on the literature [52] and compare their outcomes with the manual therapy previously adopted. The primary goal of *Neokid* is therefore to enforce remote monitoring of blood glucose values on those patients and automate the computation of the infusion rates, suggesting in real time the most appropriate values to the NICU staff. Moreover, all the neonates at the NICU are monitored and their data are displayed on a central monitoring station alerting the staff whenever an alteration of any vital parameter is noticed. This is crucial in an eventful setting, such as the NICU, where centralized monitoring facilitates the simultaneous analysis of multiple parameters and is essential for properly driving clinical decisions. Unfortunately, there is still no commercial equipment available addressing the acquisition of CGM data and making them available on the ward control panel. Before *Neokid*, CGM was accomplished through separate glucometers lying on cradle drawers and their readings could only be acquired when some member of the staff visited each cradle. Thus, *Neokid* also helps in integrating CGM data for all patients on a single PC display, tracking any clinical alteration or maneuver. Besides the plain acquisition of CGM values, *Neokid* also allows for annotating events such as rest, intraventricular hemorrage, acidosis, pneumothorax or pain and to correlate their occurrences with CGM values during data analysis.

For the *Neokid* project, the chosen CGM device is still the *Dexcom G4 Platinum with Share*. Thanks to the release by *Dexcom, Inc.* of the protocol for communicating via *Bluetooth Low Energy* with the receiver, the authors developed a telemonitoring application running on a smartphone. This application replicates in real time the information sent by the *Dexcom G4 Platinum with Share* and relays these data to the remote monitoring server using a *3G/UMTS/WiFi* connection. The server includes a web application, similar to those already discussed in the previous sections, which makes data available to nurses and doctors on a PC located in the ward room of the NICU. An additional component has also been developed to accomplish the calculations of the glucose infusion rates adopting an algorithm published in the literature [53].

**Figure 6:**
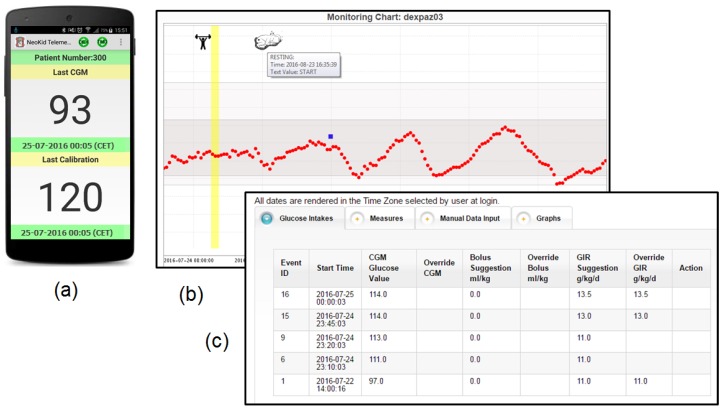
The components developed for the *Neokid* mobile application. (**a**) the smartphone application; (**b**) the detailed Continuous Glucose Monitoring track for a single patient; and (**c**) the panel with the suggested glucose infusion rates.

The functionality of the main components developed for the *Neokid* system is shown in Figure 6. The main screen of the smartphone app with the last acquired glycemia and calibration values is shown in part (a), while (b) shows the detailed CGM track for a single patient with two events. Finally, (c) illustrates the panel with the suggested glucose infusion rates.

The *Neokid* project is an interesting example of the advantages that may be achieved when software design issues enforce modularity and reusability. First, we were able to reuse the whole web application already implemented for the *AP@home* project with very limited changes. Moreover, even though the web application was mainly meant to be accessed in the ward, thanks to its generic availability on the Internet, doctors could make use of it also from their offices, and sometimes even from home. Finally, knowledge of the protocol for interacting with the CGM devices is essential, and, when it is disclosed by the manufacturers, custom applications may be successfully implemented in very short time periods.

## 5. Discussion

In this paper, we reviewed the evolution of the architectures adopted for the remote monitoring of glucose and diabetes data over the past few decades. We also illustrated our experiences in developing some telemedicine systems that were used for overseeing the operation of AP devices in adults or adolescents and for centralizing the management of blood glucose infusion rates in preterm newborns treated at the NICU. Based on those experiences, we observed that the integration of portable medical devices into remote monitoring architectures poses several issues. The first one is related with the design and development of new medical devices. We know that this process is inherently very long, mainly due to the several regulatory steps required to ensure its safe use on patients. Since, on the contrary, the evolution of ICT technologies occurs at a much faster rate, a possible solution for being successful with this integration is to take into account some sort of backward compatibility. This should be accomplished with the twofold purpose of interfacing with devices still based on old technologies and standards but quickly moving to more recent ones as soon as the devices are updated. We followed this approach exactly in our projects concerning the remote monitoring of AP devices, frequently updating the architecture over a limited time period to support its interfacing with devices using each time updated technology standards, as described in Section 4.1 and Section 4.2.

A second issue is that, differently from the ICT market, a plug-and-play approach for medical devices faces several further challenges, especially for what concerns the safety of the final system and the liability in case of errors. While, in the past, the connection among medical devices mainly occurred through wires and just required a basic serial protocol, the adoption of wireless connectivity opened up new security issues that had to be properly addressed. Security and encryption protocols are already available and regularly used in a transparent way in modern ICT applications. However, they require extensive memory and computational capabilities that are still unsuitable for resource limited devices such as CGM sensors and insulin pumps [54]. Mitigating solutions have been proposed [55], and the Diabetes Technology Society (DTS) has recently introduced a cyber-security standard (DTSec) to be adopted for securely interconnecting medical devices [56]. In the meantime, however, manufacturers have adopted solutions based on proprietary ciphers, and it will take some time before an effective standardization will actually take place. Interfacing with these devices, therefore, currently requires a close cooperation with manufacturers.

Another issue is related to the importance of taking care of the final users of a system. Once the prototype has been completed and safety issues have been addressed and verified, developers should deeply consider suggestions coming from the actual users in order to make their system more targeted to its final context of use [57]. This becomes even more important when different actors are involved, including fragile or older patients [58]. Thus, in this review, we also addressed the issue of patient’s perception. In particular, analyzing the questionnaires administered to adults during the trials held at hotels and at home, we observed that patients were concerned about being effectively safeguarded by the telemedicine service. To this aim, possible improvements might include the integration of an automatic alerting system that not only notifies the physicians about the onset of dangerous situations for the patients, but also keeps the patients themselves in the loop so that they may feel more tightly monitored and safe. Such an involvement has been experimented in the adolescents trial exploiting the AP described in Section 5, where parents highly appreciated to be included in the loop, as this enabled them to oversee the status of their children in real time.

As for the methodological standpoint, the large amount of data generated by the automatic feed of a multitude of sensors, such as those envisioned by the Internet of Things [59], is expected to exceed the investigative capabilities of a single clinical team. Addressing this issue poses several challenges, ranging from the development of new communication architectures supporting the massive usage of cloud computing [60], to the integration of heterogenous data coming from different sources [61] so that interpretation may be accomplished mostly automatically using ontology and terminology servers implemented as distributed agents [16]. Finally, on the regulatory side, many questions are still open concerning the storage and the exchange of sensitive data such as those originated by medical devices. Every country has its own regulations, and, quite often, system architectures should be adapted to comply with these. The most common hurdles are given by the ownership of medical data, the agreements to be settled with the institutions managing the servers where those data are being stored or processed, and the possibility to exchange them over public networks [62].

## 6. Conclusions

In summary, we observe that the rapid development of a large variety of affordable portable medical devices, like insulin pumps and CGM sensors, and the ubiquitous availability of networking resources, are enabling new paradigms of care in which remote monitoring becomes a key feature for enforcing safety during outpatient studies [63]. The technology also helps in the current clinical practice on chronic outpatients, as it allows remote monitoring by the caregivers (e.g., children can be monitored by their parents). Finally, the real-time acquisition of various clinical parameters and their comprehensive integration is also important to improve monitoring and accelerate treatment actions.

## Abbreviations

The following abbreviations are used in this manuscript:

3G	Third Generation Mobile Communication System
AP	Artificial Pancreas
BAN	Body Area Network
BGL	Blood Glucose Level
CGM	Continuous Glucose Monitoring
DiAs	Diabetes Assistant
DTS	Diabetes Technology Society
DTSec	Diabetes Technology Society Security Standard for Connected Diabetes Devices
GD	Gestational Diabetes Mellitus
ICT	Information and Communication Technology
MU	Medical Unit
NICU	Neonatal Intensive Care Unit
PC	Personal Computer
PDA	Personal Digital Assistant
PU	Patient Unit
SMS	Short Message Service
SOA	Service Oriented Architecture
T1D	Type 1 Diabetes Mellitus
T2D	Type 2 Diabetes Mellitus
UMTS	Universal Mobile Telecommunications System
USB	Universal Serial Bus
WAP	Wireless Application Protocol
TV	Television
